# Oxygen Evolution Reaction at IrO_2_/Ir(Ni) Film Electrodes Prepared by Galvanic Replacement and Anodization: Effect of Precursor Ni Film Thickness

**DOI:** 10.3390/molecules24112095

**Published:** 2019-06-01

**Authors:** Aikaterini Touni, Athanasios Papaderakis, Dimitrios Karfaridis, Georgios Vourlias, Sotiris Sotiropoulos

**Affiliations:** 1 Department of Chemistry, Aristotle University of Thessaloniki, 54124 Thessaloniki, Greece; thpapader@outlook.com; 2 Department of Physics, Aristotle University of Thessaloniki, 54124 Thessaloniki, Greece; dkarfari@physics.auth.gr (D.K.); gvourlia@auth.gr (G.V.)

**Keywords:** electrocatalysis, galvanic replacement, oxygen evolution, iridium dioxide, nickel

## Abstract

IrO_2_/Ir(Ni) film electrodes of variable Ni content have been prepared via a galvanic replacement method, whereby surface layers of pre-deposited Ni are replaced by Ir, followed by electrochemical anodization. Electrodeposition of Ni on a glassy carbon electrode support has been carried out at constant potential and the charge of electrodeposited Ni controlled so as to investigate the effect of precursor Ni layer thickness on the electrocatalytic activity of the corresponding IrO_2_/Ir(Ni)/GC electrodes for the oxygen evolution reaction (OER). After their preparation, these electrodes were characterized by microscopic (SEM) and spectroscopic (EDS, XPS) techniques, revealing the formation of Ir deposits on the Ni support and a thin IrO_2_ layer on their surfaces. To determine the electroactive surface area of the IrO_2_ coatings, cyclic voltammograms were recorded in the potential range between hydrogen and oxygen evolution and the charge under the anodic part of the curves, corresponding to Ir surface oxide formation, served as an indicator of the quantity of active IrO_2_ in the film. The electrocatalytic activity of the coatings for OER was investigated by current–potential curves under steady state conditions, revealing that the catalysts prepared from thinner Ni films exhibited enhanced electrocatalytic performance.

## 1. Introduction

The oxygen evolution reaction (OER) is relevant to many practical applications such as the electrolytic production of H_2_ and in processes that Dimensionally Stable Anodes (DSAs) are required. Materials with high stability and electrocatalytic activity for OER are thus sought [1]. These are composed of electrocatalytic active metal oxides on inert and stable substrates [2], replacing precious metals in their bulk form, which are no longer used in industrial applications [2,3]. IrO_2_ is known to be the state-of-the-art catalyst for OER, as it requires low overpotential for OER and provides good corrosion resistance and stability in acidic solutions [4]. In order to prolong IrO_2_ lifetime titanium is commonly used as a substrate, because it is a chemically inert and inexpensive support material [1]. Recently OER anodes based on Ir have been developed using Ni [5,6] as an additional component since Ni has been reported to improve the electrocatalytic performance of IrO_2_ for OER [1,5,7,8]. Ni and Co single or mixed oxide or sulfide compounds are also known to show increased OER activity in alkaline media [9,10,11] and this is attributed to their high conductivity and electrochemical stability as well as to synergistic interactions between catalyst components.

Sotiropoulos and co-workers have employed Ni widely as a galvanic replacement intermediate/ precursor to improve precious metal performance and utilization for methanol oxidation [12,13,14] and the hydrogen [15] and oxygen [7] evolution reactions. Taking advantage of Ni negative standard potential in comparison to that of precious metals (i.e., Pt, Ir, Ru), they employed spontaneous deposition of precious metals on Ni via the galvanic replacement method. Galvanic replacement of Ni by a more precious metal (e.g., Pt, Ir, Ru) is thermodynamically favorable due to the positive difference between the standard potential of the two half-reactions (1) and (2) [16], resulting in the case of Ir to the overall reaction of (3):

IrCl_6_^2−^ + 4e^−^ ⇌ Ir + 6Cl^−^ ^0^ = +0.860 V (vs. SHE) (1)

Ni^2+^ + 2e^−^ ⇌ Ni E^0^ = −0.257 V (vs. SHE) (2)

*x*Ni + IrCl_6_^2−^ → Ir(Ni*_x_*_−2_) + 2Ni^2+^ + 6Cl^−^ (3)

Due to the superior electrocatalytic performance of IrO_2_/Ir(Ni) film catalysts for OER [7,17] (an improvement to IrO_2_ well-established properties and activity [18,19,20,21]), the aim of this work has been to investigate the effect of the thickness of the precursor nickel layer on the morphology, composition and electrochemical behavior of IrO_2_/Ir(Ni) electrodes prepared by the galvanic replacement of Ni by Ir, followed by anodization. The significance of this report arises from the fact that catalyst electrochemical performance depends on catalyst composition and morphology and both are expected to be affected by Ni thickness. For this reason IrO_2_/Ir(Ni)/GC electrodes with different quantity/ thickness of precursor Ni were investigated, as formed by electrodeposition of Ni on glassy carbon, GC, substrates at constant potential. Based on SEM microscopy and XPS spectroscopy as well as on current-potential curves, a structure/composition-property relationship between the bimetallic catalyst and its OER activity has been sought.

## 2. Results and Discussion

### 2.1. Microscopic and Spectroscopic Characterization of IrO_2_/Ir(Ni)/GC Catalyst Film Electrodes

**Figure 1:**
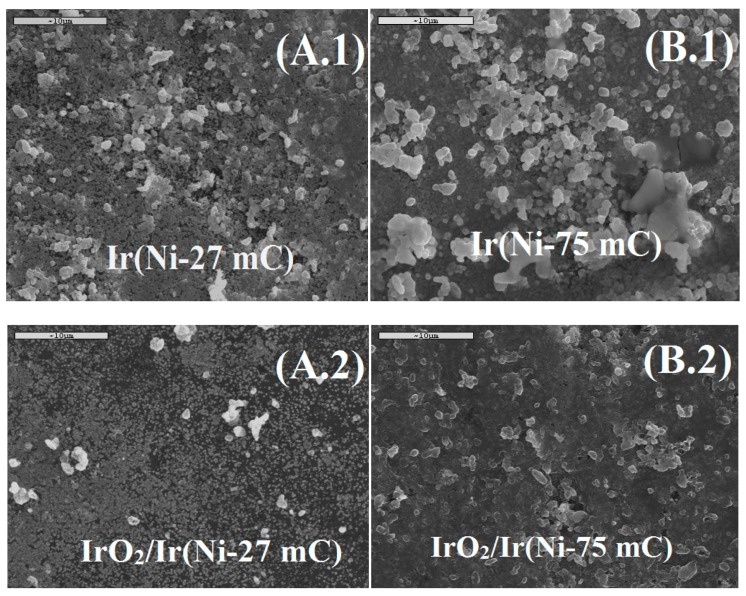
(A.1, B.1): SEM micrographs of Ir(Ni)/GC samples prepared by galvanic replacement of (A.1) 27 mC (382 mC cm^−2^) and (B.1) 75 mC (1062 mC cm^−2^) electrodeposited Ni. (A.2, B.2): SEM micrographs of the corresponding IrO_2_/Ir(Ni)/GC catalysts prepared by anodization of the samples shown in A.1, B.1 respectively. (Scale bars correspond to 10 μm).

SEM micrographs depict the morphology of the catalyst surface and, combined with EDS analysis, confirm the presence of Ir deposits on Ni. Figure 1(A.1,B.1) present the surface morphology of “27 mC” and “75 mC” Ir(Ni)/GC samples, prepared by Ir galvanic deposition on 27 and 75 mC electrodeposited Ni films (136 and 363 nm thick) on a GC substrate. Figure 1(A.2,B.2) present the corresponding IrO_2_/Ir(Ni)/GC catalysts prepared by subsequent anodization of the samples shown in A.1 and B.1 (by 10 potential sweep cycles at 50 mV·s^−1^ between −0.30 and +1.15 V vs. SCE-see next sub-section). Brighter areas are attributed to thick Ir or IrO_2_/Ir particles, grey areas to thinly-coated Ni areas or passive Ni oxides whereas dark areas are linked to parts of the GC substrate that have been revealed during the replacement and/or anodization process. Larger Ir particles seem to form on the “75 mC’’-thicker Ni precursor sample and a thinner and more porous film results from the “25 mC”-thinner Ni precursor sample. Overall, there is good coverage of the surface without a dramatic change when passing from as-prepared ((A.1), (B.1)) to anodized samples ((A.2), (B.2)); the latter observation indicates fast Ir deposition and significant Ni protection-coverage by Ir and is more pronounced in the case of the thin film (fewer changes in morphology upon anodization) when compared to the thicker film (where some large Ir particles seem to collapse, because of neighboring Ni areas dissolving during the electrochemical treatment).

Table 1 below presents the elemental Ir and Ni EDS analysis of the samples shown in Figure 1.

It can be seen that for both thin (27 mC) and thick (75 mC) Ni precursor films, the non-anodized/ as-prepared Ir-coated catalysts have a higher Ni content, which decreased upon anodization since the remaining non-coated metallic Ni undergoes active dissolution and its surface oxides are also subject to partial trans-passive dissolution at the highest positive potentials used; only Ni areas protected-covered by Ir are guaranteed to survive and retain a significant part of their metallic character after the electrochemical treatment. Also, as expected for a non-surface sensitive technique such as EDS which probes the entire, thinner than 1 μm, film, it has been confirmed that the “75 mC” electrodes have a much higher Ni content than the “27 mC” ones.

The anodized “27 mC” and “75 mC” samples were also examined by XPS analysis (Table 2 and Table 3), a more surface sensitive technique in comparison to EDS analysis that can also provide at the same time information about the oxidation state of elements. According to Table 1, based on wide area binding energy range scans (not shown), in the case of “27 mC”, XPS analysis gives 93.18% Ir and 6.28% Ni (total) atom concentration for as received/non-Ar-sputtered samples (0 nm of material sputtered-off, denoted hereafter as “0 nm”). The measurable increase in Ir (total) concentration recorded by XPS in comparison to that given by EDS (the former being ca. 93% the latter 77%) is in line with Ir segregation on the surface and an Ir-rich shell structure (as expected for the galvanic replacement mechanism). The Ir/Ni atom composition ratio remains almost unchanged within the first layers of the film as the gradually Ar-sputtered samples have atomic compositions of 92.68% Ir-7.32% Ni (at 1 nm depth) and 93.35% Ir-6.65% Ni (at 3 nm depth), indicating a thicker than 3 nm Ir-rich shell. The “75 mC” sample showed a notably higher Ni content in the surface layers (e.g., 51.66% at 0 nm) that fluctuated in depth; this indicates a more incomplete Ni coverage by Ir and/or thinner remaining Ir films, pointing to a more irregular replacement process in the case of thicker deposits (in line with the morphological differences shown in Figure 1).

**Figure 2:**
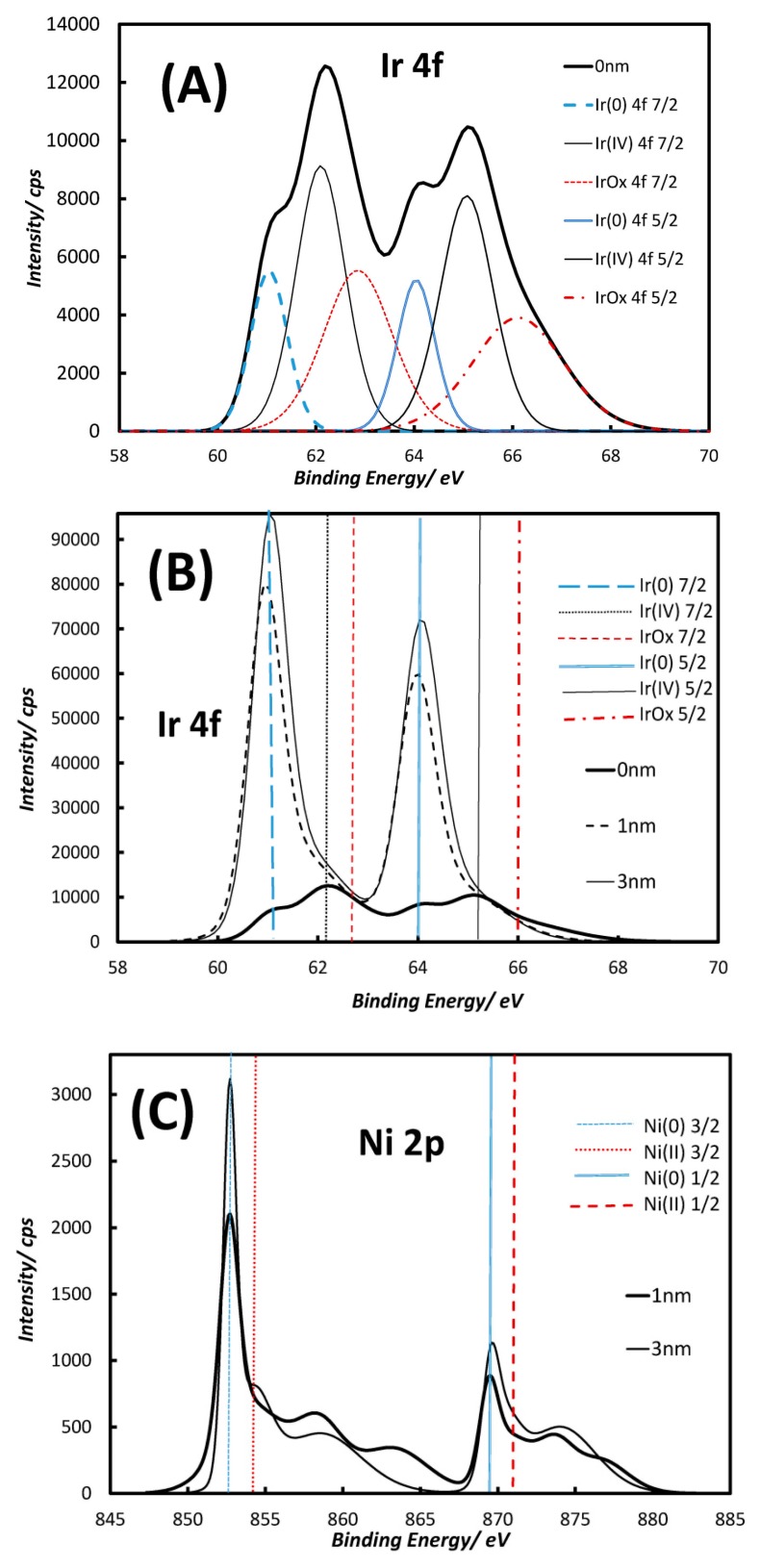
XPS spectra of “27 mC” IrO_2_/Ir(Ni)/GC samples. (**A**) Ir 4f photoelectrons (non-sputtered sample, analysis at a 0 nm depth); (**B**) Ir 4f photoelectrons (non-sputtered and sputtered samples, analysis at 0, 1 and 3 nm depths); (**C**) Ni 2p photoelectrons (sputtered samples, analysis at 1 and 3 nm depths). The vertical lines show the binding energy positions for various oxidation states of Ir or Ni.

Figure 2 depicts the narrow energy range slow XPS scans at a depth of 0 nm that provide information for the oxidation state of Ir and Ni.

The peaks at ca. 61.0 (4f _7/2_) and 64.0 eV (4f _5/2_) can be attributed to metallic Ir(0) [22], while those at 61.9 (4f _7/2_) and 64.9 eV (4f _5/2_) to amorphous hydrous oxides of Ir(IV) [19,22,23,24,25,26,27]. Finally, although the peaks at binding energies of 62.8 and 66.0 eV have not been unanimously distributed to a particular state, they are usually assigned to hydrous Ir oxides in which Ir shows up with many oxidation states Based on the relevant literature the latter couple of peaks can be attributed to Ir(III) [28,29,30], Ir(IV) satellites [29,30,31,32,33,34], or even to higher oxidation states [34,35] such as Ir(V) [36] and Ir(VI) [18,31,37]. However Ir(III) and Ir(VI) are mostly observed when IrO_2_ is thermally deposited on a substrate from IrCl_3_ salts, in which case it either remains as Ir(III) (whereby assignment of Ir(III) to binding energies higher than Ir(IV) is due to the Ir-Cl bond) or it is converted to Ir(VI) due to high temperature [38]. This is not a plausible scenario for our catalysts as they are prepared via galvanic replacement at moderate temperatures followed by electrochemical anodization. Furthermore, Ni 3p is also reported at these binding energies [17,21,39,40,41,42,43,44], but in our case, since at 0 nm a Ni 2p signal could not be detected (and it is therefore not shown in Figure 2C), Ni 3p should not either be observed because its intensity is expected to be weaker than that of Ni 2p. Hence, for the catalysts of this work, we believe that it is more possible that the peaks recorded at 62.8 and 66.0 eV are due to either surface Ir(V) [36] (as this signal is only recorded at the surface (0 nm) and then disappears) or to Ir(IV) satellites. Figure 2B presents the XPS spectra evolution as one moves from the surface (0 nm) to a 3 nm depth of the coating. It can be seen that IrO_2_ resides mainly on a very thin outer shell less than 1 nm thick and it is mainly in its metallic form as one moves into the core of the coating, in full agreement with the proposed mechanism of Ir metal galvanic deposition and subsequent anodization to IrO_2_. Finally Figure 2C presents XPS spectra for Ni 2p. (For the unsputtered samples, i.e., at 0 nm depth, a very weak and noisy Ni 2p signal has been recorded (particularly for the “27 mC” sample), indicating significant (or full) coverage of underlying Ni by IrO_2_, and is thus not shown). The couple of peaks at binding energies of 852.7 and 869.5 eV is attributed to metallic Ni [45,46,47] and that at 854.2 and 870.7 eV to Ni(II) in the form of NiO [45,47,48,49]. Additionally, satellite peaks in the range of 850–880 eV characteristic of oxides [50] show up. A very similar XPS picture was obtained for the thick nickel precursor sample of “75 mC”.

Quantitative analysis of the atomic concentration ratio of metallic-to-total Ir (Ir/(Ir + IrO_x_) as a function of depth shown in Table 2 and Table 3 (from data of Figures similar to 2A) shows that metallic Ir increases across the deposit, indicating the existence of metallic Ir in the core. At the same time the presence of IrO_2_ oxide drops sharply as one moves inside the film (from 19.7 and 13.6% at 0 nm to 61.9 and 54.4% at 1 nm), confirming the existence of very thin IrO_x_ films. Another interesting point that should be made based on the data of the last column of Table 2 and Table 3 is that Ni oxide can be found at considerable levels (ca. 25–60%) together with metallic Ni well inside the film. This may be explained by the fact that IrO_2_ prepared by the anodization of the Ir shell is known to be nanoporous thus permitting electrolyte access to Ni underlayers which are in turn oxidized. Note that it is indeed the presence of Ni oxides (and their partial leaching) that has been proposed to increase the OER activity of IrO_2_/Ni catalysts by disrupting the Ir-O-Ir bonds [17].

### 2.2. Surface Electrochemistry of the Electrodes

**Figure 3:**
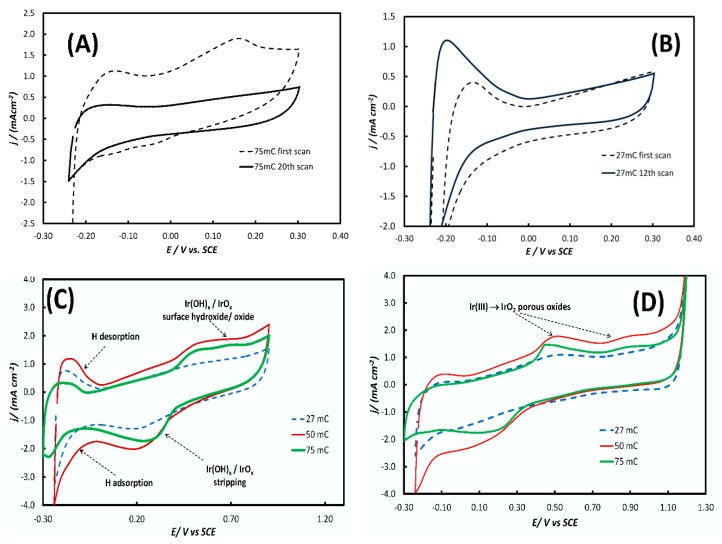
Cyclic voltamograms of the IrO_2_/Ir(Ni)/GC electrodes prepared by galvanic replacement followed by anodization, recorded at a potential sweep rate of 50 mV s^−1^ up to +1.2 V, in a deaerated 0.1 M HClO_4_ solution. Current is normalized per electrode substrate geometric area. The solid line curves of (**A**) and (**B**) and all curves of (**C**) and (**D**) correspond to stabilized scans.

Prior to anodization, the “27 mC”, “50 mC” and “75 mC” samples, as received from the galvanic replacement process, underwent cyclic voltammetry in a limited potential range to confirm the presence of metallic Ir by its typical Ir surface electrochemistry and dissolve/passivate any unreacted surface Ni. Hence, the Ir-Ni/GC electrodes were scanned in a deaerated solution of 0.1 M HClO_4_ at a voltage scan rate of 50 mV·s^−1^ in the potential range of (a) underpotential adsorption/desorption of a hydrogen monolayer/submonolayer on Ir starting at ca. −0.10 V (Figure 3A,B) and (b) reversible Ir surface oxide/hydroxide formation and reduction at ca. +0.55 V/+0.30 V respectively (Figure 3C).

In the case of the thick Ni precursor sample of “75 mC” (Figure 3A), large anodic currents are recorded (around +0.20 V) during the very first scan and can be attributed to the dissolution and/or passivation of uncovered surface Ni [51,52] (in accordance with the high Ni content of the surface as indicated by the XPS results of Table 2 and Table 3). On the contrary, no anodic currents in the Ni oxidation range were recorded for the thin “27 mC” sample, indicating good surface coverage by Ir. A stabilized voltammetric picture was typically obtained after more than 10 scans. Extending the positive potential limit to +0.9 V (Figure 3C), one can notice the formation of a surface IrO*_x_*/Ir(OH)*_x_* oxide/hydroxide (at ca. 0.55 V) and its quasi-reversible stripping-reduction at ca. +0.25 V during the reverse scan. In the same Figure the peaks associated with H adsorption/desorption can now be clearly seen due to surface activation/cleaning through surface oxide formation and stripping. The typical (see for example [15,17,18,19,20]) metallic Ir surface electrochemistry shown in Figure 3C is macroscopic proof that Ir is deposited and stays as a metal on Ni, according to the galvanic deposition mechanism proposed.

Scanning the potential up to +1.2 V vs. SCE (Figure 3D), Ir is gradually converted to stable, three-dimensional, porous Ir(IV) oxides (see for example [7,53]). In our samples, oxide growth stops and the voltamograms stabilize after typically at least 10 scans. These Ir(IV) oxides grow into the bulk of Ir deposits and form porous oxides which are known to be effective for OER [17,18,19,54,55]. At E > +1.1V Ir(IV) is converted to Ir(V) and Ir(VI) and OER starts to occur. Anodization is achieved through potential cycling between hydrogen and oxygen evolution at a potential scan rate of 50 mV·s^−1^ until voltamograms stabilization (approximately 10 scans). During anodization the peaks attributed to H adsorption/desorption are suppressed, which is evidence that metallic Ir has been converted to Ir oxides on the surface. These results are in line with [7,17,18,19,20,53,54,55].

The charge under the anodic part of the voltamograms in the entire 0.0–+1.1 V range is often used as an indicator of IrO*_x_* quantity or, more accurately, electroactive surface area [7,14,54,56]. Figure 3D depicts the stabilized voltamograms when the electrodes had been exposed to a maximum anodic potential of +1.20 V but not to higher potentials where OER is studied (see next section).

**Scheme 1:**
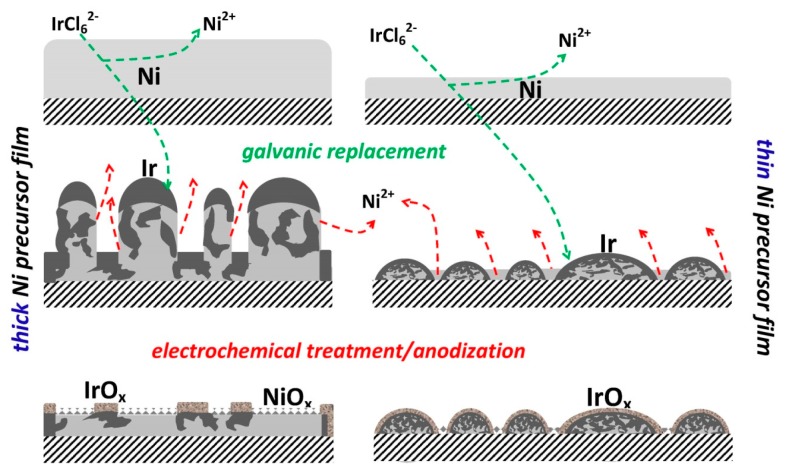
Proposed mechanism for film morphology and composition evolution during the galvanic replacement of Ni layers by Ir, followed by electrochemical treatment and anodization, for thin and thick Ni precursor films.

The variation of the charge below the curves of the voltammograms of Figure 3 (which is indicative of IrO_2_ electroactive area and quantity) with precursor Ni thickness can be explained as follows. The thinner the initial Ni deposit the more efficient Ni protection-coverage by Ir. On the contrary, in the case of thick Ni deposits (see SEM micrographs in Figure 1 as well as the discussion in Section 2.1 and Scheme 1 below), the large Ir particles formed on the Ni surface collapse, due to the dissolution of neighboring uncovered Ni areas during electrochemical treatment (these areas are more extended for thicker films). Hence the total catalyst amount on the electrode (and its electroactive area/charge for oxide formation/ stripping as estimated from the voltammograms of Figure 3) is the result of two opposite trends as the thickness of Ni increases: a rise in reactant (Ni) availability for Ir deposition and increased rates for Ir removal due to uncovered Ni dissolution upon the subsequent anodization treatment. This interplay leads to the appearance of a maximum in the surface area-charge vs. initial Ni quantity relationship, corresponding to the “50 mC” sample (with the voltammetry curve of the thicker sample of “70 mC’’ lying slightly below that of the “50 mC” but higher than that of the “27 mC” sample).

**Figure 4:**
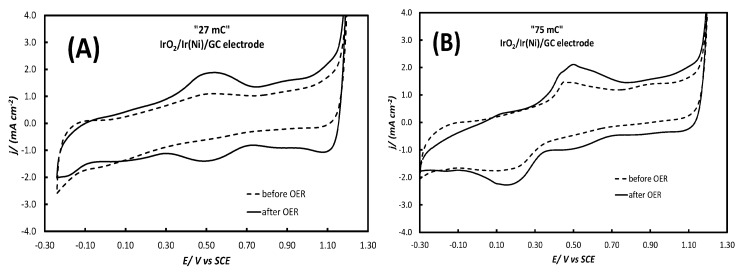
Cyclic voltamograms of the IrO_2_/Ir(Ni)/GC electrodes ((**A**): “27 mC” electrode; (**B**): “75 mC” electrode) prepared by galvanic replacement followed by anodization, recorded at a potential sweep rate of 50 mV s^−1^ up to +1.20 V, in a deaerated 0.1 M HClO_4_ solution. Dashed line curves correspond to stabilized scans prior to OER experiments while solid line curves to stabilized scans after OER experiments at potentials higher than +1.20 V.

Figure 4A,B show that there is change/activation of the electroactive surface area during the occurrence of OER and this should be taken into account if currents are to be normalized per IrO_2_ charge (see next section). The estimated IrO_2_ charge for the three electrodes studied, before and after OER, is given in Table 4 below. The surface roughening/activation of the electrode after exposure to relatively high potentials may be attributed to electro-dissolution of Ni sites that did not react during the galvanic replacement process and/or were passivated in the subsequent potential cycling experiments. Furthermore, the dissolution of the partially protective Ir hydrous layer (formed on the surface of the electrode during the initial potentiodynamic scans [18]) after exposure within the OER potential range, may facilitate the oxidation of deeper Ir layers, resulting in an expansion of the oxide network and thus an increase of electroactive surface area.

### 2.3. Oxygen Evolution Reaction (OER)

The detailed protocol for the OER study is described in the *Materials and Methods* section below. Briefly, a rotating disc IrO_2_/Ir(Ni)-modified GC electrode, RDE, (rotated at 1500 rpm) has been used to remove O_2_ bubbles evolving during OER and to keep any film of O_2_ formed (that may act as a diffusion barrier) thin and reproducible (in case the process enters a mixed kinetic/mass transfer regime). OER has been studied at constant potential and the steady-state current (typically having settled after 200 s) has been recorded. Also, cyclic voltamograms in the −0.25–+1.20 V range have been recorded between OER experiments to account for electroactive active area changes.

**Figure 5:**
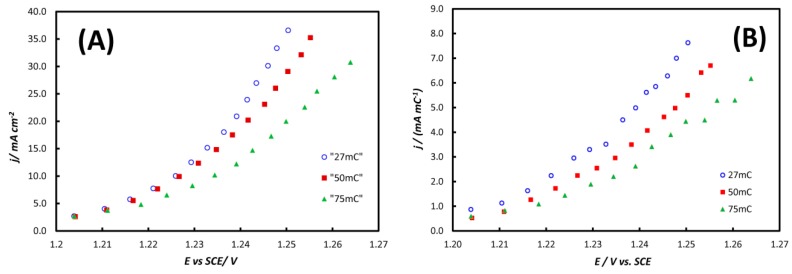
Current density vs applied potential steady state data for oxygen evolution reaction (OER) at IrO_2_/Ir(Ni)/GC electrodes in 0.1 M HClO_4_ solution. Current densities are presented per (**A**) substrate geometric area and (**B**) Ir oxide formation charge.

Figure 5A,B below present the steady-state current density vs applied potential, corrected for the uncompensated resistance, for three IrO_2_/Ir(Ni)/GC electrodes of different Ni-precursor film thicknesses (“27 mC”, “50 mC”, “75 mC”). The current density is normalized both per electrode geometric area (A) and the charge of the anodically grown IrO_2_ (B), the latter being representative of the electroactive area of porous IrO_2_.

Figure 5A shows that the “27 mC” (thinner Ni precursor film) gives similar currents with the “50 mC” one but higher currents than the “75 mC” electrode (thicker Ni precursor film). More important, according to Figure 5B, the IrO_2_/Ir(Ni) catalyst prepared from the thinner Ni-precursor film shows superior intrinsic electrocatalytic activity for OER. The origin of this effect should be linked to the different overall Ni content of the thin and thick Ni precursor samples since EDS data of Table 1 give a 22.9% Ni content for the “27 mC” coating compared to the much higher 67.1% of the “75 mC” sample (note that the best mixed Ir-Ni oxide catalysts of [17] seem to stabilize during OER to a low, 12% at Ni content). More important, the higher activity of the “27 mC” catalyst may also be associated to its rich in IrO_2_ surface (XPS data of Table 2 give 93.18% at Ir for the “27 mC” sample as opposed to 48.34% for the “75 mC” sample).

**Figure 6:**
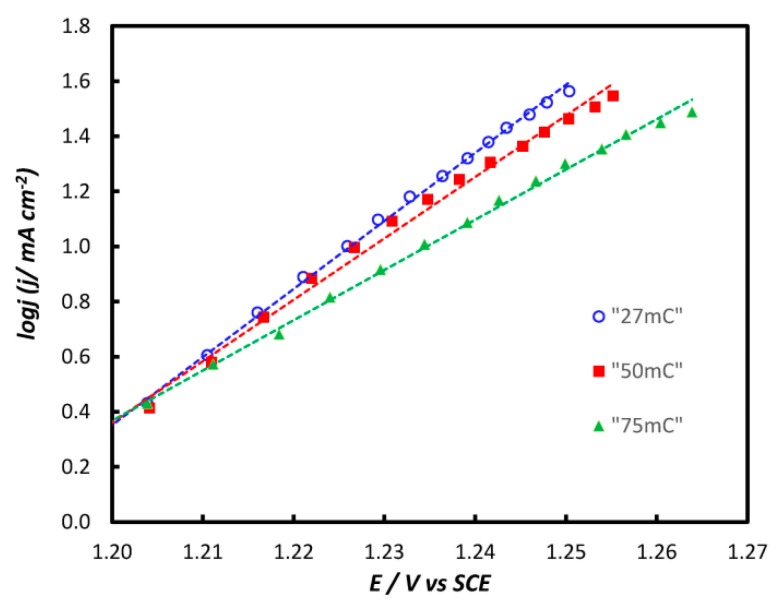
Tafel plots for OER with applied potential corrected for the uncompensated resistance. Current is normalized per geometric area.

Figure 6 presents the Tafel analysis of the data presented in Figure 5A above.

The corresponding Tafel plots have slopes of 40, 45 and 55 mV·dec^−1^ for ”27 mC”, “50 mC” and “75 mC” IrO_2_/Ir(Ni)/GC electrodes respectively. These values of Tafel slopes are common for IrO_2_ catalysts (40–60 mV·dec^−1^) [7,54,57,58].

Finally, Table 5 below shows that the IrO_2_/Ir(Ni)/GC electrodes of this work present comparable and even enhanced electrocatalytic activity with respect to other IrO_2_-Ni and IrO_2_/Ir electrodes prepared both by similar or different methods [7,14,53,55], whether the current is normalized per substrate geometric area (indicative of overall activity) or the IrO_x_ formation charge (indicative of intrinsic electrocatalytic activity).

## 3. Materials and Methods

### 3.1. Preparation of IrO_2_/Ir(Ni)/GC Electrodes

The preparation of IrO_2_/Ir(Ni)/GC electrodes involves three basic steps: Ni electrodeposition on GC (at 382, 707, 1062 mC cm^−2^ charge densities; 27, 50 and 75 mC passed from the 0.071 cm^2^ of the disc electrode substrate), galvanic replacement of Ni by Ir and electrochemical anodization to form IrO_2_ oxides on its surface. These electrodes have been referred to as “27 mC”, “50 mC” and “75 mC” respectively based on the quantity of the deposited Ni. Ni was electrodeposited on a freshly grinded (600-grit emery paper) GC electrode (3 mm disc RDE GC, Metrohm) in a deaerated aqueous solution of 0.01 M nickel sulfamate ((H_2_NSO_3_)_2_Ni·4H_2_O, 99%, Fluka, Honeywell, Charlotte, NC, USA), 0.227 mM nickel chloride (NiCl_2_, puriss > 97%, Merck, Burlington, MA, USA) and 0.025 M boric acid (H_3_BO_3_, puriss 98%, Merck). During the potentiostatic (E = −1.1V vs. SCE) deposition, the cathodic charge was recorded by chronoamperometry–chronocoulometry to control Ni deposition. Ni thickness was calculated based on Faraday’s law, assuming (a) a 100% Ni deposition current efficiency (as evidenced by separate gravimetric experiments that gave current efficiency values higher than 98%), (b) minimal porosity of Ni (as evidenced by SEM pictures of similar Ni deposits [59] and (c) taking into account the density of Ni (d_Ni_ = 8.9 g cm^−3^). A thickness of 136, 242 and 363 nm was estimated. Then the Ni/GC electrode was rinsed with HCl (to remove any native Ni surface oxides) and directly immersed into a deaerated solution of 10^−3^ M K_2_IrCl_6_ + 10^−3^ M HCl at 65 °C for 45 min (potassium hexachloroiridate, 99.99% trace metals basis, Aldrich, St. Louis, MO, USA; hydrochloric acid 37% for laboratory use, Chem-Lab, Zedelgem, Belgium) for galvanic replacement to take place [7] and form Ir(Ni)/GC. During the galvanic replacement process a stream of N_2_ was maintained over the cell ensuring an oxygen-free blanket atmosphere. All solutions used were freshly prepared. Finally, the Ir(Ni)/GC electrode underwent electrochemical anodization to form Ir oxides. Anodization was achieved by scanning the Ir(Ni)/GC electrode multiple times via the cyclic voltammetry technique in the potential range between hydrogen and oxygen evolution (−0.30 and +1.20 V vs. SCE in 0.1 M HClO_4_) to form porous, open-structure Ir oxides, which are known to exhibit excellent electrocatalytic activity for OER [7,18,19,20,21].

### 3.2. Electrochemical Setup and Procedures

All the electrochemical measurements were performed at room temperature in a deaerated 0.1 M HClO_4_ (perchloric acid 70% Merck) solution using a GC rotating disc electrode (RDE, Eco Chemie, Utrecht, The Netherlands). The Ir(Ni)/GC electrode (working electrode) was placed in the central chamber of a three-compartment glass cell at a very small distance from the luggin capillary of the chamber containing a KCl-saturated calomel reference electrode (SCE). A Pt-foil, placed in the third chamber, separated from the central one with a glass frit, was used as the counter electrode. All potentials mentioned in this article are referred to SCE (saturated in KCl). Electrochemical measurements including cyclic voltammetry (CV) and chronoamperometry (CA) were carried out with the help of an Autolab PGSTAT302N (Eco Chemie, Utrecht, The Netherlands) workstation, which was controlled via NOVA 1.11.2 software (Eco Chemie, Utrecht, The Netherlands).

Stabilized cyclic voltamograms of the Ir(Ni)/GC electrode were recorded at a potential scan rate of 50 mV·s^−1^ in 0.1 M HClO_4_ deaerated solution: (a) within the hydrogen adsorption/desorption potential range (between −0.3 and +0.3 V) to ensure the presence of metallic Ir on Ni during the first, galvanic replacement, step and to dissolve/passivate uncovered Ni, (b) within the reversible Ir surface oxide formation potential range (between −0.3 V and +0.9 V) to observe the typical Ir surface electrochemistry and (c) up to the onset of the oxygen evolution reaction process (up to 1.2 V) to form hydrous bulk porous Ir oxides (electrochemical anodization).

To study OER, constant potential chronoamperometric measurements were carried out according to the above protocol: (a) apply the current interrupt method to estimate the uncompensated resistance (aiming at correcting the potential values), (b) pulse the potential to the value of interest (onset and beyond of OER) and record the current for 200 s (until current stabilizes to achieve steady-state conditions) with the simultaneous electrode rotation at 1500 rpm (in order to remove O_2_ bubbles and to keep any diffusion barrier formed due to oxygen evolution constant, in order to reach steady state conditions) and (c) scan the electrode at 50 mV·s^−1^ for 4 cycles up to 1.2 V (to condition the electrode surface and record any changes in the electroactive area via the charge associated with the recorded surface electrochemistry-IrOx oxide formation). This protocol was repeated for each potential value from 1.20 V to 1.34 V in 10 mV intervals.

### 3.3. Microscopic and Spectroscopic Characterization

A JEOL 6300 microscope (JEOL Ltd, Akishima, Japan) equipped with an Oxford ISIS 2000 X-ray EDS (EDAX) system (Oxford Instruments Ltd, Oxford, UK) was used to obtain SEM micrographs and EDS elemental composition analysis. XPS measurements of (IrO_2_/Ir-TiO_2_)/Ti samples were performed at an AXIS Ultra DLD X-ray Photoelectron Spectrometer by Kratos Analytical, Wharfside, Manchester, UK, with a monochromated Al-Ka1 X-ray beam (λKa = 1.4866 Å). A 4 keV Ar^+^ ion beam helped to (a) remove adventitious carbon and other surface contaminants from the samples and (b) etch the samples at a specific depth (20 s of sputtering results in a ca. 1 nm material etching).

## 4. Conclusions

### 4.1. IrO_2_/Ir(Ni) Catalyst Morphological, Compositional and Electrochemical Characterization; Mechanism of Catalyst Preparation

SEM microscopy revealed that Ir is deposited by galvanic replacement of Ni as larger particles on the thicker Ni precursor film, indicating that metallic Ir deposition becomes uneven as the time of the galvanic replacement process is prolonged due to the presence of large Ni quantities available to be exchanged/chemically etched into the solution.

XPS analysis showed the presence of a very thin (thickness < 1 nm) IrO_2_ film on the surface of the catalyst. The catalyst prepared on a thin Ni precursor film has a surface composition that is more than 90% at Ir whereas that of a thicker (almost by three times) film is less than 70% at Ir.

The surface electrochemistry of the catalysts, as obtained by cyclic voltammetry, revealed that Ir is initially in its metallic form and it is converted to IrO_2_ oxides by anodization at potentials more positive than +1.20 V. Also, the as prepared Ir(Ni) films from thick Ni precursor deposits exhibited high anodic currents during their first exposure to anodic potentials pointing to the presence of large uncovered Ni areas that have been subsequently electrochemically dissolved or passivated.

All of the above findings may be explained by the mechanism presented in Scheme 1 below.

### 4.2. Catalyst Performance as an OER Anode

The amount of precursor Ni deposit affects Ni content at the surface of the deposits and as a result its effect on IrO_2_ OER catalytic properties. It has been found that the thinner the precursor deposit the lower the Ni/NiO*_x_* presence at the surface and the higher the intrinsic catalytic activity for OER. From the catalysts tested, the best performance was observed for a catalyst originating from a ca. 136 nm thick Ni film and having a final bulk composition of ca. 88% at Ir-12% at Ni and a surface composition of ca. 93% Ir-7% at Ni.

It has thus been proved that the structure and performance of OER catalysts prepared by galvanic replacement of Ni by Ir can be tuned by controlling the quantity of the former.

## Figures and Tables

**Table 1:** EDS results (relative atomic concentration percentage of Ir and Ni) for as-prepared (Ir(Ni)) and anodized (IrO_2_/Ir(Ni)) “27 mC” and “75 mC” films.

Electrodes	Ir/% at	Ni/% at
“27 mC” as prepared	77.1 (±1.3)	22.9 (±1.3)
“27 mC” anodized	87.8 (±1.0)	12.2 (±1.0)
“75 mC” as prepared	5.3 (±1.8)	94.7 (±1.8)
“75 mC” anodized	32.9 (±7.2)	67.1 (±7.2)

**Table 2:** Sputter-etch XPS analysis of the anodized “27 mC” sample based on wide area binding energy range scans (first two composition columns) and narrow energy range scans (last two composition ratio columns).

Sputter-Etch Depth/nm	Ir(total)/% at	Ni(total)/% at	Ir(0)/Ir(total)	Ni(0)/Ni(total)
0	93.18	6.82	19.7	n/a (very poor Ni signal)
1	92.68	7.32	61.9	43.36
3	93.35	6.65	68.6	58.14

**Table 3:** Sputter-etch XPS analysis of the anodized “75 mC” sample based on wide area binding energy range scans (first two composition columns) and narrow energy range scans (last two composition ratio columns).

Sputter-Etch Depth/nm	Ir(total)/% at	Ni(total)/% at	Ir(0)/Ir(total)	Ni(0)/Ni(total)
0	48.34	51.66	13.6	0
1	70.67	29.33	54.4	75.7
3	62.4	37.6	65.3	60.3

**Table 4:** Charge density corresponding to IrO_2_ anodic growth, as estimated from the integration of the anodic part of cyclic voltamograms in deaerated 0.1 M HClO_4_ solutions between 0.0 and +1.2 V.

Electrode	q_IrO2_/mC cm^−2^ (before OER)	q_IrO2_/mC cm^−2^ (after OER)
“27 mC”	2.82	5.17
“50 mC”	4.77	5.40
“75 mC”	4.18	6.76

**Table 5:** OER performance of IrO_2_/Ni catalysts reported in this work and in indicative literature as quantified by the overpotential, η, required to achieve a given area and mass specific current density or, conversely, the values of the latter at a given overpotential.

Catalyst	*j = 10 mA·cm^−2^*	*j = 0.5 mA mC^−1^*	*η = 300 mV*	
	η/mV	η/mV	j/mA cm^−2^	j/mA mC^−1^
Electrochemically anodized IrO_2_-Ni film via galvanic deposition (707 mC cm^−2^ electrodeposited Ni) [7]	360	354	1	0.05
Thermally prepared mixed Ir-Ni oxides (varied Ni content) [17]	200–370	290–315	1–7	0.33–0.85
Electrochemically anodized bulk Ir (300 cycles) [56]	390	301	9.7	0.47
This work (varied Ni content)	299–307	253–270	9–11	1.8–3
